# An Investigation of Three-Finger Toxin—nAChR Interactions through Rosetta Protein Docking

**DOI:** 10.3390/toxins12090598

**Published:** 2020-09-16

**Authors:** Alican Gulsevin, Jens Meiler

**Affiliations:** 1 Department of Chemistry, Vanderbilt University, Nashville, TN 37212, USA; jens.meiler@vanderbilt.edu; 2 Institute for Drug Discovery, Leipzig University Medical School, 04103 Leipzig, Germany

**Keywords:** nicotinic acetylcholine receptors (nAChR), three-finger toxins (3FTX), acetylcholine binding protein (AChBP), protein–protein docking, computational modeling

## Abstract

Three-finger toxins (3FTX) are a group of peptides that affect multiple receptor types. One group of proteins affected by 3FTX are nicotinic acetylcholine receptors (nAChR). Structural information on how neurotoxins interact with nAChR is limited and is confined to a small group of neurotoxins. Therefore, in silico methods are valuable in understanding the interactions between 3FTX and different nAChR subtypes, but there are no established protocols to model 3FTX–nAChR interactions. We followed a homology modeling and protein docking protocol to address this issue and tested its success on three different systems. First, neurotoxin peptides co-crystallized with acetylcholine binding protein (AChBP) were re-docked to assess whether Rosetta protein–protein docking can reproduce the native poses. Second, experimental data on peptide binding to AChBP was used to test whether the docking protocol can qualitatively distinguish AChBP-binders from non-binders. Finally, we docked eight peptides with known α7 and muscle-type nAChR binding properties to test whether the protocol can explain the differential activities of the peptides at the two receptor subtypes. Overall, the docking protocol predicted the qualitative and some specific aspects of 3FTX binding to nAChR with reasonable success and shed light on unknown aspects of 3FTX binding to different receptor subtypes.

## 1. Introduction

### 1.1. Nicotinic Acetylcholine Receptors (nAChR) Play Important Roles 

Nicotinic acetylcholine receptors (nAChR) are pentameric ligand-gated ion channels belonging to the Cys-loop receptor superfamily. nAChR play important roles in neuromuscular transmission, addiction, nociception, and cognition [1]. The traditional ligand binding site of nAChR is called the orthosteric site. This site is characterized by a number of aromatic residues that form an “aromatic cage”. Ligands bind to that cage through cation–π and hydrophobic interactions [2,3]. Heteromeric nAChR such as the muscle-type and α4β2 nAChR have two orthosteric binding sites formed by α and non-α subunits [4], whereas homomeric nAChR such as α7 and α9 nAChR have five putative binding sites at the α-α interfaces [5].

### 1.2. Three-Finger Toxins (3FTX) Target nAChR

**Figure 1:**
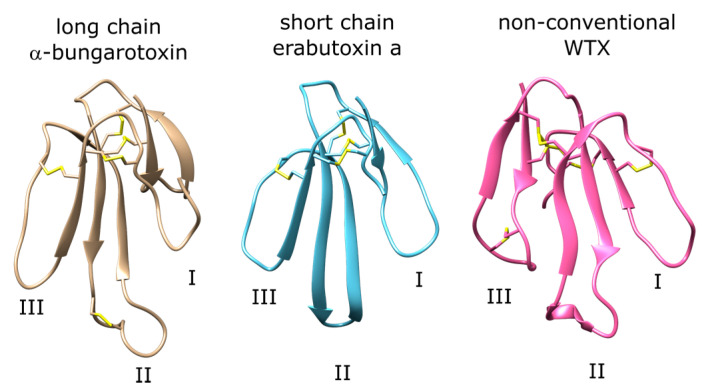
Examples of long-chain, short-chain, and non-conventional three-finger toxins (3FTX) with the three loop regions labeled. The disulfide bonds are shown explicitly with sulfur atoms shown with yellow.

One group of peptide ligands that target nAChR are three-finger toxins (3FTX). 3FTX are found in many species including *Elapid* and *Hydrophiid* snakes. These are among the major toxins that cause envenomation through binding at nAChR at the postsynaptic junction. 3FTX are formed by three parallel β-sheets and contain four or five disulfide bonds. 3FTX fall under the main categories of long-chain, short-chain, and non-conventional 3FTX [6]. Long-chain 3FTX consist of 66–75 residues and have five disulfide bonds (Figure 1, left panel). One of these disulfide bonds constrain the loop II of the peptide, which forms a short helical domain that binds to the aromatic cage of the nAChR orthosteric site formed by tyrosine and tryptophan residues. Short-chain 3FTX consist of 60–62 residues and lack the fifth disulfide bond that forms the helical motif at loop II observed in long-chain 3FTX (Figure 1, middle panel). Instead, a β-hairpin is observed at the tip of the peptide, and this region interacts with the orthosteric site residues [7]. Non-conventional 3FTX may have different lengths and disulfide bonding patterns compared to the long- and short-chain 3FTX (Figure 1, right panel). Their selectivity profiles and structural features vary from peptide to peptide. 

### 1.3. 3FTX Interact with Different nAChR Subtypes with Varying Selectivity

Although the general properties of the nAChR orthosteric sites are similar, and the aromatic cage is conserved throughout nAChR, different venom peptides have different selectivity profiles for nAChR subtypes [8]. Both long- and short-chain 3FTX can bind to muscle-type nAChR with high affinity, whereas only long-chain 3FTX bind to neuronal-type α7 and α4β2 nAChR with high affinity in general. Non-conventional neurotoxin candoxin and its derivatives [9,10] and some dimeric short-chain α-neurotoxins such as haditoxin [11] and fulditoxin [12] can bind to both neuronal and muscle-type nAChR with high affinity. Monomeric short-chain 3FTX can bind to both muscle-type and neuronal nAChR, but their affinity towards the former is larger than the latter [13]. Understanding the selectivity profile of 3FTX is important both for the treatment of their toxic effects and to develop pharmacological tools targeting the nAChR in a selective manner. 

### 1.4. Structural Information from AChBP Can Be Used to Model Peptide–nAChR Interactions

Experimental binding studies with nAChR are challenging. There can be issues associated with stability and expression of nAChR, which limits the number of available structures for different nAChR subtypes. Another group of proteins used to study the nAChR is acetylcholine binding proteins (AChBP) due to properties that are better amenable to experimental studies [14]. AChBP is the common name given to a number of soluble proteins typically secreted by molluscan species [15,16]. AChBP have a homo-pentameric structure that resemble the homomeric α7 nAChR with 21–24% residue homology. The quaternary structure of the AChBP consists of the same domains and the general scaffold as the nAChR, whereby the important loops for ligand binding including the C- and F-loops are structurally conserved. Ever since the release of the first AChBP structure [17], they have been used as a platform to study ligand binding to nAChR, and multiple crystal structures of nAChR ligands in complex with AChBP are available in the Protein Data Bank [14].

Although there are multiple structures of α-conotoxins co-crystallized with nAChR homologs, there is a shortage of structures co-crystallized with long- and short-chain 3FTX. Currently, only muscle-type *Torpedo* nAChR [18], α-bungarotoxin bound to an α7ECD–AChBP chimera (α7-AChBP) and monomers of α1 and α9 [19,20,21], and α-cobratoxin bound to AChBP [22] are available in the Protein Data Bank (PDB) [23]. 

Further, while the AChBP structures are useful to understand the general interactions between a peptide and nAChR, they fail to explain subtype-specific interactions of 3FTX with nAChRs. Therefore, a method that can be used to 3FTX–nAChR interactions starting from sequence or structure is necessary to obtain structural information on these systems. Modeling studies on 3FTX thus far shed light on the binding properties and orientations of different 3FTX including WTX, NTII, candoxin, bucandin, and κ-bungarotoxin [24,25,26]. However, there is no established protocol so far that was shown to yield good binding orientations for a diverse group of 3FTX. 

### 1.5. Rosetta Peptide Docking Can Be Used to Identify 3FTX–nAChR Interactions

Rosetta peptide docking protocol ToxDock was previously developed to model α-conotoxin–nAChR interactions through an ensemble docking approach with good success [27]. Adapting the ToxDock protocol for 3FTX, we utilized the Rosetta protein–protein docking application [28] that can be used to predict the binding properties of venom peptides to nAChR. First, eleven AChBP and α7-AChBP experimental structures with bound neurotoxins were obtained from the PDB. The peptides in these crystal structures were re-docked into their native proteins and cross-docked into two randomly selected AChBP structures in the test set to assess the success of Rosetta in reproducing the geometries observed in the native structures. Additionally, cross-docking studies can confirm that success of this docking protocol is robust with respect to minor backbone conformational changes in the starting structures. Second, a different series of 15 long- and short-chain 3FTX for which qualitative AChBP binding information exists were docked into the α-bungarotoxin-bound AChBP crystal structure to test whether the protocol can distinguish between the binder and non-binder peptides. Lastly, peptides with known selectivity profiles were docked into α7 and the two different interfaces of the muscle-type *Torpedo* nAChR (αγ and αδ) to test whether the protocol can be used to explain the selectivity differences of nAChR-targeting venom peptides. 

## 2. Results

### 2.1. 3FTX and α-Conotoxin Structures Were Considered for the Re-Docking and Cross-Docking Calculations

The peptides bound to AChBP and homolog structures covered α-bungarotoxin, α-cobratoxin, and α-conotoxin derivatives PnIA, ImI, TxIA (A10L), BuIA, GIC, PeIA, LsIA, LvIA (Table 1). α-conotoxin derivatives have different structures compared to 3FTX proteins limiting their usability as a benchmark. However, the benchmark with α-conotoxins serves two important purposes that are consistent with the application of the protocol to 3FTX. The first one is that the putative mode of interaction of both, α-conotoxins and 3FTX, involve similar contacts, whereby a positively charged residue interacts with the aromatic cage of the receptor, and other peptide residues form accessory interactions within the orthosteric site. By re-docking the peptides into the corresponding protein structures, we test whether the Rosetta score function is capable of accurately scoring the interactions between these peptides and AChBP. The second purpose of the α-conotoxin docking calculations is to see whether the protocol is capable of sampling the conformational changes associated with peptide binding. nAChR have flexible C- and F-loops that may go through large structural changes upon ligand binding, and the cross-docking calculations address whether the ensemble docking protocol we chose is able to reflect these structural changes.

Each peptide in the test set was re-docked to its corresponding protein structure. In addition, each peptide was docked into one or two other randomly selected AChBP structures belonging to the same species (*Aplysia californica* or *Lymnaea stagnalis*) to assess whether the protocol we followed is robust enough to remodel the orthosteric site configurations observed in AChBP structures with different peptides bound.

### 2.2. The Docking Protocol Consists of Peptide–Protein Complex Relaxing and Protein–Protein Docking Steps

**Figure 2:**
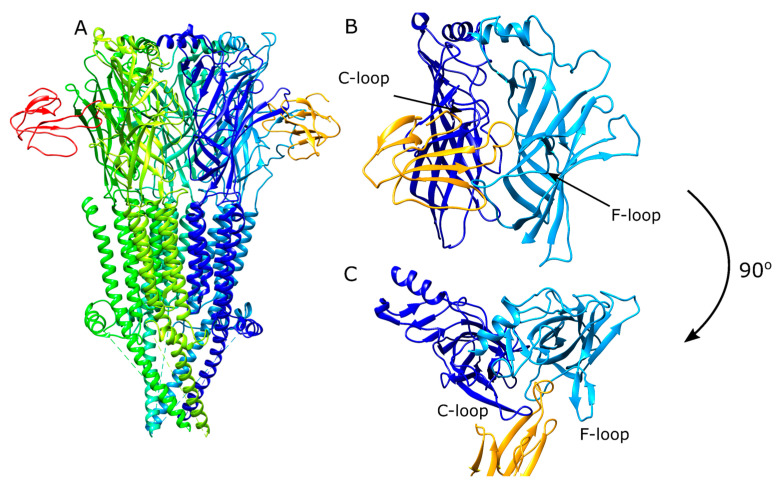
Structure of the α-bungarotoxin-bound *Torpedo* nicotinic acetylcholine receptors (nAChR) (**A**) (PDB ID: 6UWZ) and its αδ subunit ECD viewed from side (**B**) and top (**C**). The C- and F-loops are labeled on the ECD figures.

The modified ToxDock protocol we followed consists of three main steps. Shortly, the structures from the PDB database were first subjected to a fixed-backbone relax to relieve any energetic frustrations. Next, 200 relax trajectories were run to optimize the interactions between the peptides and the proteins and to allow movement of the C- and F-loop (Figure 2) in response to peptide binding. Five lowest-scoring structures from this step were used as the input for docking. A total of 500 peptide docking calculations were done for each input structure to a total of 2500 docked structures. Values such as total score, interface score, total root mean square deviation (RMSD), interface RMSD, and contact recovery were calculated for the assessment of the performance of the docking protocol. 

Finally, the score versus RMSD plots were inspected to determine whether they display an “energy funnel” whereby the scores of the generated structures converge to a single, native-like low-energy pose. These plots are commonly used for docking calculations and are useful tools to see whether there is a single low-energy configuration or multiple low-energy configurations belonging to the same peptide–protein complex. When comparing the docked poses with native structures, the lack of funneling is suggestive of issues with scoring or sampling. In other cases, the lack of a well-defined energy funnel is indicative of lack of stable binding, although individual bound poses may be still observed. Because a visual inspection is not always sufficient to decide whether a funnel is present, goodness-of-energy-funnel (P_near_) values [36] were calculated in addition. 

### 2.3. Rosetta Re-Docking Protocol Predicts Binding Conformation of the Peptides with RMSD Values Less than 2 Å 

**Figure 3:**
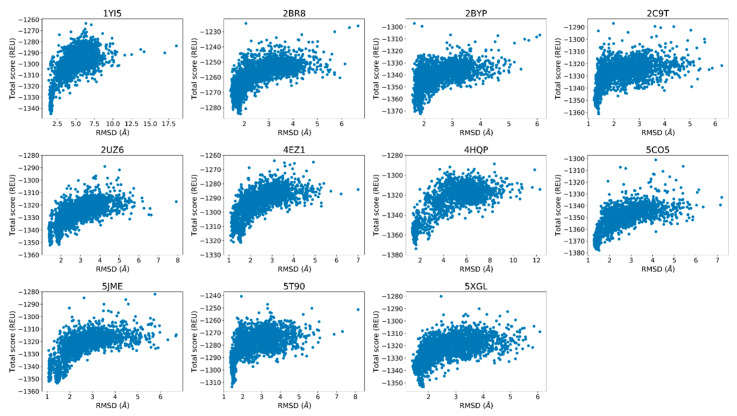
The score versus root mean square deviation (RMSD) plots of the re-docking calculations using the native crystal structures as the reference.

The top five poses selected based on lowest interface scores for each docking calculation showed significant similarities with each other and the native structures. The score versus total RMSD plots displayed a “energy funnel”, i.e., with lower RMSD the Rosetta energy also improves, in support of a stable binding pose for the peptides (Figure 3). The average RMSD value calculated for the top five poses were less than 2.0 Å (1.6 ± 0.2 Å) within a range of 1.3 Å and 1.9 Å (Table S1). The ligand geometries showed positional shifting up to 3.0 Å, which was caused by the changes in the C- and F-loop conformations during the relax calculations (Figure S1). 

### 2.4. Fraction of Native Contacts Conserved Indicate Rosetta’s Success in Predicting Side-Chain Conformations 

We also looked at the fraction of native contacts conserved (Fnat) to determine whether the sidechain contacts predicted by Rosetta are consistent with the native contacts. Fnat is the fraction of the predicted residue-residue contacts with respect to the residue–residue contacts calculated for the native structure, whereby a residue–residue contact is defined as atoms of any two residues being closer than 5 Å. The Fnat values were between 0.63 and 0.91 overall with an average of 0.78. The lowest Fnat value was observed for α-conotoxin-LsIA-bound AChBP and the highest Fnat was observed for the α-bungarotoxin-bound α7-AChBP structure. 

There was no obvious correlation between Fnat and structural parameters such as side-chain rotamer outliers, R-values, or structure resolution (Figure S2). On the other hand, some experimental structures showed slight variations in the coordinates of the bound peptide molecules at different interfaces despite the pseudo-symmetric nature of the AChBP complexes. This suggests that there is some plasticity in the interaction. The differences in the C-loop and F-loop coordinates belonging to the same structure can be seen in Figure S3. Overall, the reduced Fnat values and higher RMSDs of docking poses could be attributed to this apparent breathing of the complex rather than ambiguities caused by structural defects in the starting structures. 

### 2.5. Cross-Docking Calculations Yield Slightly Diminished Accuracy Compared to the Native Re-Docking Calculations

A possible caveat of using the native protein structures for the re-docking calculations is imprinting structural information that bias the docking calculations. One way of overcoming this problem is “cross-docking” based on the use of protein structures with different ligands bound in their native state. In order to test whether the success observed for the native re-docking calculations apply to docking to other AChBP structures, we ran cross-docking calculations with these proteins and calculated the RMSD values. Of the eleven structures used for the analyses, one is an α7-AChBP chimera, two are from *Lymnaea stagnalis*, and eight are from *Aplysia californica* (Table 1). The α7-AChBP structure (4HQP) was not cross-docked due to absence of another such protein structure. The two peptides bound to *Lymnaea stagnalis* AChBP were docked to the AChBP of each other (1YI5 and 5T90). For the remaining eight structures, two other *Aplysia californica* AChBP structures were randomly selected and cross-docking calculations were done using these proteins.

The RMSD values of the cross-docking calculations were higher compared to the native re-docking calculations due to variations in the C-loop and F-loop configurations for some peptides (Table S2). On the other hand, similar results were observed for the majority of the peptides, suggesting small variations in the configurations of these two loops are tolerated by the protocol. The largest RMSD values were calculated for the 1YI5/5T90 cross-docking pair. This large difference may be caused by docking of a long-chain neurotoxin into an AChBP structure co-crystallized with an α-conotoxin, which have significant structural differences. 

Based on the overall success of Rosetta in reproducing the native conformations of the peptide–AChBP complexes, we tested its performance in predicting the binding properties of fifteen long and short-chain 3FTX to AChBP. 

### 2.6. Experimental AChBP Binding Data Was Used to Test the Success of the Docking Protocol

In a recent study by Albulescu et al., mass-spectrometry-based fishing experiments with AChBP tested binding of 17 snake venoms from different species [37]. In these experiments, four long-chain 3FTX bound to AChBP, whereas thirteen short-chain and non-conventional 3FTX showed no binding. We focused on these peptides in our analyses and applied our docking protocol to test whether the binding patterns calculated through docking are consistent with the experimental results. Specifically, we looked for peptide poses that interact with the aromatic cage residues of the AChBP and sought for funneling behavior of the score versus RMSD plots that are indicative of a single stable binding pose for the proteins. 

Of the 17 venom peptides selected initially (Table S3), two had putative structures significantly different than the 3FTX motif (**T11** and **T13**), so these molecules were excluded since it was not possible to place them in the binding site in a manner consistent with other peptides. Therefore, a total of 15 peptides were used for the docking calculations. The sequences of these peptides can be found in Table S4. A bound peptide was defined as a peptide that formed at least one interaction with the AChBP aromatic cage residues (Y186, Y193, Y91, Y53, and W145) and partly resided under the C-loop. In addition, convergence of the score versus RMSD plots to an unambiguous low-energy pose was considered to be a sign of predicted binders since non-binders are not expected to have a single stable binding pose.

### 2.7. The Docking Protocol Accurately Predicts the Binding Properties of Long-Chain 3FTX to AChBP

**Figure 4:**
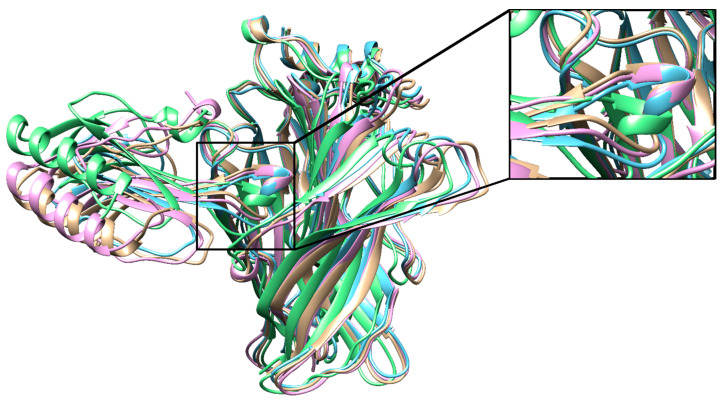
The lowest-scoring binding poses of T01 (pink), T02 (blue), T03 (green), T04 (beige) aligned on each other with a close-up of the loop II region.

All four binding peptides docked into the orthosteric site similar to the long-chain 3-FTXs α-bungarotoxin and α-cobratoxin, in contact with the aromatic cage of the protein. This is not surprising considering that these four peptides are 81–100% homologous to the α-cobratoxin structure at their overlapping regions. The only difference was the binding pose of **T03**, which stood ~2 Å below the remaining three peptides due to its slightly more closed C-loop (Figure 4). The most important interaction formed between these peptides and AChBP was between a conserved arginine residue plus a conserved aspartate residue and Y184. Y184 was previously shown to be the most important contributor to α-bungarotoxin–α7-AChBP interactions [19,38]. The score versus RMSD plots of all four peptides showed funneling behavior indicative of a low-scoring stable binding pose (Figure S4, top row). 

### 2.8. Most Non-Binders Interact Only with the Outer Face of the C-Loop and have No Significant Interactions with the Aromatic Cage Residues

For the 11 non-binders in the experiments, 10 peptides docked outside the aromatic cage and one had weak interactions with some aromatic residues. There was some tendency to form a funnel only in the score versus RMSD plots of **T06**, **T10**, **T14**, and **T16**, but these funnels were less well defined when compared to that of the **T01**–**T04** (Figure S4). P_near_ values were calculated as a metric of the funneling quality as described elsewhere [36]. Shortly, it is the weighted probability of finding decoys near the native-like state. P_near_ values close to 1 indicate funneling with a unique low-energy conformation, and values close to 0 indicate the lack of a well-defined funnel with possible low-energy alternative poses. The calculated P_near_ values were 0.93, 0.99, 0.99, and 0.75 for the four peptides, respectively, in support of funneling behavior.

For **T06** and **T10**, the main interactions were between the N-terminus β-hairpin of the protein and the outer surface of the C-loop (Figure S5). For **T14**, the first and second β-hairpins split apart and interacted with the outer face of the C-loop on both sides in a clamp-like manner. The only peptide that bound near the aromatic cage was **T16**, which interacted with AChBP through the positively charged R32 and D30 that formed an indirect hydrogen bond with (+)Y188 through (−)Y164 whereby (+) stands for the principal subunit bearing the C-loop forming the orthosteric site and (−) stands for the complementary subunit forming the other half of the binding interface. 

Overall, the docking protocol was successful in distinguishing AChBP-binding peptides from non-binders. Next, 3FTX with known nAChR binding properties were docked to α7, and muscle-type nAChR models investigate whether the binding affinities of these peptides can be distinguished by the docking protocol. 

### 2.9. Peptides with Diverse Properties Were Docked to α7 and Muscle-Type nAChR to Identify Interactions Associated with Binding

A total of eight ligands (Table S5) with diverse properties and known binding affinities or IC_50_ values were docked to a homology model of the α7 nAChR ECD and to the αγ and αδ interfaces of the newly released muscle-type *Torpedo* nAChR ((α_1_)_2_βγδ) structure [18]. Of these ligands, α-bungarotoxin and α-cobratoxin are long-chain 3FTX that bind to α7 and muscle-type nAChR with high affinity. The native muscle-type nAChR structure has two α-bungarotoxin molecules bound; therefore, the α-bungarotoxin docking calculations were run as additional controls to assess Rosetta’s success in predicting the binding orientations at the nAChR. 

Drysdalin is a long-chain 3FTX that inhibits ligand binding to muscle-type and α7 nAChR with nanomolar IC_50_ values [39]. Candoxin is a short-chain 3FTX antagonist of both the muscle-type and α7 nAChR with nanomolar IC_50_ values [9]. Erabutoxin-a is a short-chain 3FTX that binds the muscle-type nAChR with nanomolar IC_50_ values but α7 nAChR with micromolar IC_50_ values [13]. SCNTX and Pr-SNTX are short-chain 3FTX that bind to muscle-type nAChR. There is no data regarding whether SCNTX binds to α7 nAChR, whereas Pr-SNTX has no antagonist activity on α7 nAChR at concentrations up to 10 μM [40,41]. Oh9-1 is an ω-bungarotoxin that lacks the positively charged amino acids conserved among α-neurotoxins, yet it binds the muscle-type nAChR with no binding to α7 nAChR up to 100 μM concentration [42]. 

For all the peptides, the top five docking poses were selected based on lowest interface scores, and these poses were analyzed. The residue–residue interactions were determined based on visual inspection and per-residue energy breakdown calculations done with Rosetta. 

### 2.10. α-Bungarotoxin and α-Cobratoxin Poses Are Consistent with Homologous Crystal Structures and Mutagenesis Experiments with Slight Differences

**Figure 5:**
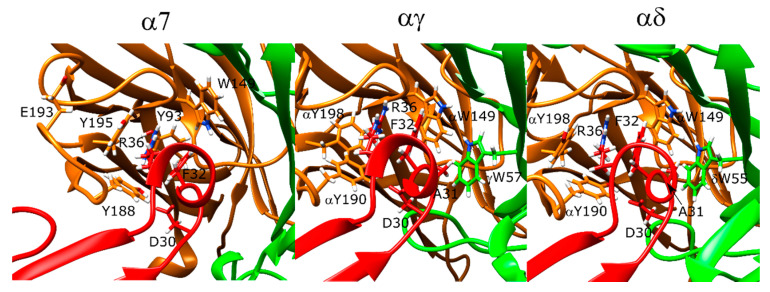
Structures of the lowest-scoring α-bungarotoxin-nAChR complexes at the α7 (**left**), αγ (**middle**), and αδ interfaces (**right**). The orange color stands for the positive subunit, the green color stands for the negative (complementary) subunit, and the red color stands for the peptide.

The lowest-scoring binding orientation of α-bungarotoxin at the α7 nAChR showed marked similarities to the α-bungarotoxin-bound α7-AChBP structure (PDB ID: 4HQP). The F32 residue of the peptide formed π–π stacking interactions with the R36 residue of the peptide, which sat between the protein residues Y188, Y195, Y93, and W149. There was also a hydrogen bond between R36 and Y93 -OH group. Another positively charged residue K38 formed a salt bridge with E189. In addition to these interactions, the negatively charged D30 residues formed a hydrogen bond with Y188 (Figure 5, left).

In the *Torpedo* nAChR docking calculations, there were slight variations between the experimental structures and the docking results. On the other hand, α-bungarotoxin binding poses to the αγ and αδ interfaces showed marked similarities with each other and with the α7 nAChR poses (Figure 5, middle and right panels). The position of the α-bungarotoxin molecule was ~3 Å different than the native *Torpedo* nAChR structure due to changes at the C- and F-loops at both αγ and αδ interfaces at the relax stage. On the other hand, conformation of R36 and its interaction partners were similar and the hydrogen bond between D30 and αY190 was retained. R36 had π–π stacking interactions with the peptide residue F32, and these two residues were in the middle of the aromatic residues αY190, αY198, and αW149 that were shown to be important for α-bungarotoxin binding in experimental studies [43,44,45]. D30 also formed a hydrogen bond with αY190. F32 formed hydrophobic interactions with δL121 (γL119), and A31 formed hydrophobic interactions with δW57 (γW55) again known to be important for α-bungarotoxin binding [46]. Interestingly, neither the experimental structure nor the docking models showed interactions between K51 or K52 and the protein as suggested by NMR experiments [43].

For α-cobratoxin, there were several differences between the α-cobratoxin-bound AChBP structure and our docking results. In the AChBP crystal structure, R33 extends into the aromatic cage and forms interactions with Y192 (α7 Y195) and W143 (α7 W149). There is also a hydrogen bond between D27 and Y188. 

**Figure 6:**
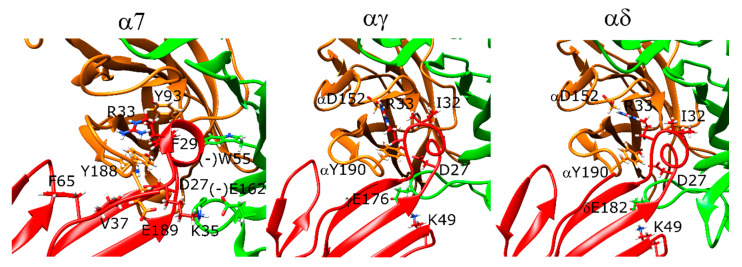
Structures of the lowest-scoring α-cobratoxin-nAChR complexes at the α7 (**left**), αγ (**middle**), and αδ interfaces (**right**). The orange color stands for the positive subunit, the green color stands for the negative (complementary) subunit, and the red color stands for the peptide.

In the lowest-scoring docking poses of α-cobratoxin at α7 nAChR, R33 interacted with Y188, but not with the other two aromatic residues. Instead, F29 formed hydrophobic interactions with Y93 and (−)W55. Next to Y188, F65 and V37 formed hydrophobic interactions with F187. K35 formed a salt bridge with (−)E162. The N-terminal peptide residues T6 and P7 formed interactions with the outer face of the C-loop. Moreover, R68 was close to the negatively charged E189 of the receptor. There were no interactions between D27 and Y188 in the α-bungarotoxin experimental structure, but instead, there was a weak salt bridge between D27 and R186 (Figure 6, left panel). 

At the αγ and αδ interfaces of the muscle-type nAChR, R33 extended above the C-loop and formed direct interactions only with αD152 and αY190 (Figure 6, middle and right panels). The space between the aromatic cage residues was instead filled with F32 due to tilting of the α-cobratoxin molecule compared to the α7 nAChR case. D27 formed a hydrogen bond with αY190. In addition to the interactions similar to that of α7 nAChR, K49 formed a salt bridge with δE182 and γE176, correspondingly, which poses one of the most significant differences between α-cobratoxin binding to α7 and muscle-type nAChR [47].

### 2.11. Drysdalin Behaves Differently than α-Cobratoxin and α-Bungarotoxin Due to Conformational Differences

Although a long-chain 3FTX, drysdalin binding was different than α-bungarotoxin and α-cobratoxin binding due to conformational differences caused by differences in its sequence. Drysdalin lacks five of the eight conserved amino acids among the long-chain 3FTX, yet it acts as an antagonist of α7 and muscle-type nAChR [39]. 

**Figure 7:**
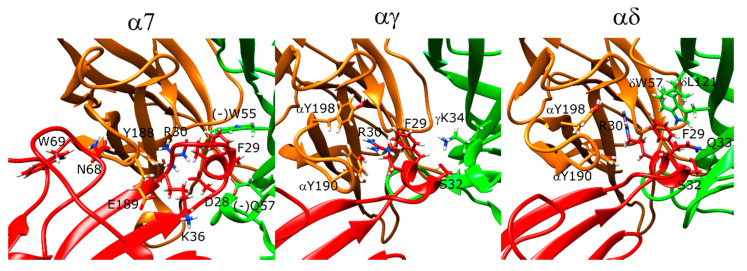
Structures of the lowest-scoring drysdalin-nAChR complexes at the α7 (**left**), αγ (**middle**), and αδ interfaces (**right**). The orange color stands for the positive subunit, the green color stands for the negative (complementary) subunit, and the red color stands for the peptide.

Homology modeling of drysdalin yielded models with different loop II conformations with respect to the rest of the peptide, suggesting a mobile nature for this region. The lowest-scoring conformation was used for the docking calculations. The non-conserved arginine residue that plays a role in interacting with aromatic residues (R30) was below that of its conserved equivalents in α-bungarotoxin and α-cobratoxin structures by ~5 Å in α7 calculations. The different positioning of R30 resulted in this residue extending towards below the C-loop forming a hydrogen bond with Y188 and a salt bridge with the peptide residue D28. F29 formed hydrophobic interactions with the (−)W55 and backbone interactions with (−)D164. Moreover, K36 formed a salt bridge with E189 (Figure 7, left panel). Another different feature of drysdalin was the involvement of its long C-terminus in interactions with the receptor structure. Drysdalin residues N68 and W69 formed interactions with the outer face of the C-loop, consistent with the decrease in the activity of the C-terminus truncated drysdalin in experimental studies [39]. 

At the αγ and αδ interfaces, R30 interacted with αY190 and αY198. F29 formed hydrophobic interactions with δW57 and δL121, but the distance between these residues and F29 was longer at the αγ interface (Figure 7, middle and right panels). S32 formed a hydrogen bond with the peptide residue Q33 and γK34, correspondingly, at αδ and αγ interfaces. The interactions between the peptide residues N68 and the protein observed for α7 were also observed for these two interfaces. 

### 2.12. Candoxin Binds α7 and Muscle-Type nAChR Differently

**Figure 8:**
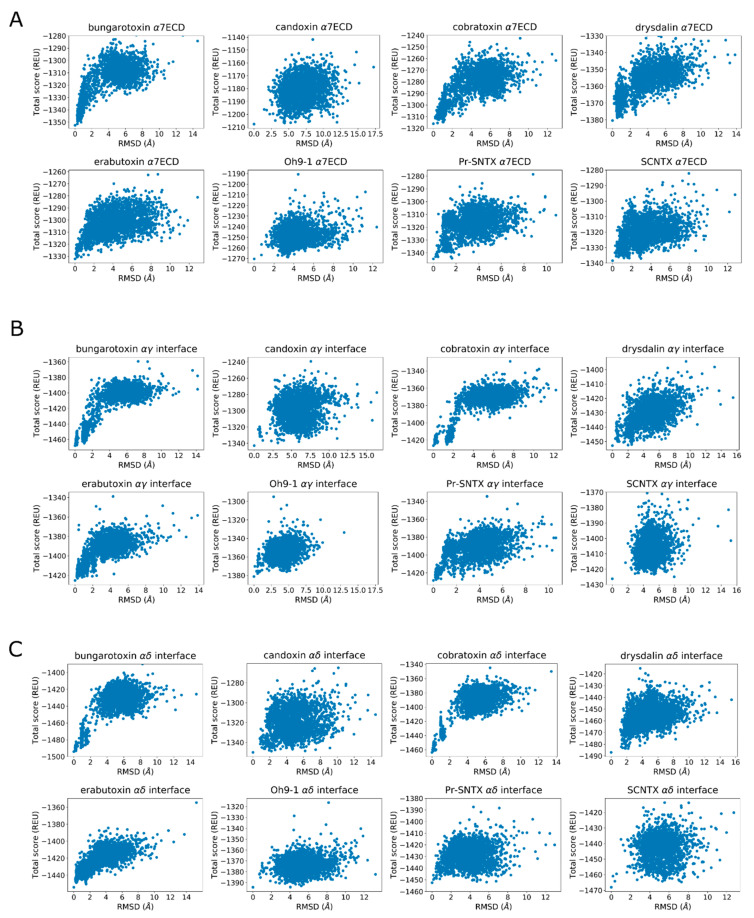
Score versus RMSD plots calculated for the eight peptides at the (**A**) α7 interface, (**B**) αγ interface, (**C**) αδ interface.

**Figure 9:**
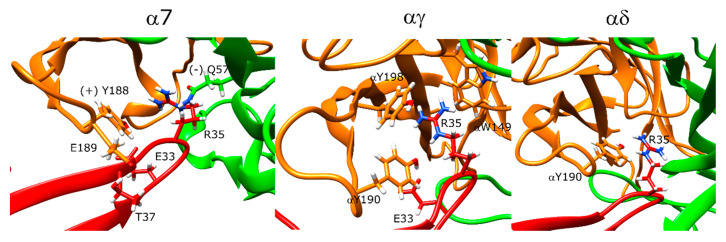
Structures of the lowest-scoring candoxin-nAChR complexes at the α7 (**left**), αγ (**middle**), and αδ interfaces (**right**). The orange color stands for the positive subunit, the green color stands for the negative (complementary) subunit, and the red color stands for the peptide.

Candoxin poses at the α7 nAChR and muscle-type nAChR interfaces did not yield a single stable conformation as seen by the scattered values in the score versus RMSD plots (Figure 8A–C). However, the top five poses showed similarities at these interfaces and these interactions were analyzed. In α7, the strongest interaction between the peptide and the receptor as measured by the per-residue energy breakdowns was a hydrogen bond between T37 and E189. R35 faced the bottom side of the C-loop and only had weak interactions with Y188. The stronger interactions of this residue were with (−)Q57 through hydrogen bonding. E33 formed a hydrogen bond with Y188 (Figure 9, left panel). On the other side of the C-loop, R38 formed an interaction with Y188, similar to R35. In addition to these interactions, E33 formed a hydrogen bond with Y188. Residues such as N7 and R12 on the N-terminus also formed interactions with the outer section of the C-loop. 

At the αγ interface, candoxin bound below the C-loop and the R35 residue extended above to interact with αY190, αY198, and αW149 (Figure 9, middle panel). E33 formed a hydrogen bond with αY190. Instead of the histidine and phenylalanine residues most short-chain 3FTX have between the positively charged arginine and negatively charged aspartate/glutamate residues, candoxin had an alanine which interacted with the β-carbon of the γT36 residue. Moreover, R32 formed a salt bridge with γE164. At the αδ interface, the C-loop was mostly closed, which placed the candoxin molecule outside the orthosteric site, and there were no significant interactions between candoxin and the orthosteric residues of the αδ interface (Figure 9, right panel). 

### 2.13. Erabutoxin-a Interacts with α7 and Muscle-Type nAChR

**Figure 10:**
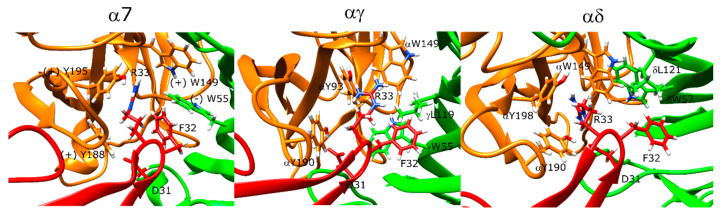
Structures of the lowest-scoring erabutoxin-a-nAChR complexes at the α7 (**left**), αγ (**middle**), and αδ interfaces (**right**). The orange color stands for the positive subunit, the green color stands for the negative (complementary) subunit, and the red color stands for the negative (complementary) subunit, and the red color stands for the peptide.

In the α7 nAChR docking calculations, erabutoxin-a poses interacted with the α7 ECD through three main residues: D31, F32, and R33 on the very tip of the β-hairpin structure that partially entered the orthosteric site (Figure 10, left panel). The erabutoxin-a poses formed interactions with the aromatic cage residues Y188, Y195, and W149 through R33. D31 also formed a hydrogen bond with Y188. The peptide residue F32 formed hydrophobic interactions with (−)W55. On the outer side of the C-loop, there were several strong interactions between the peptide and the receptor. T35 formed a hydrogen bond with E189. Moreover, N-terminal residues 7–11 formed interactions with the outer side of the C-loop.

At the αγ interface, R33 formed interactions with αY190, αY93, and αW149 (Figure 10, middle panel) whereas the αY93 interaction was replaced with αY198 at the αδ interface. D31 formed a hydrogen bond with αY190. F32 formed hydrophobic interactions with δL121 and δW57, and γL119 and γW55 (Figure 10, right panel). In addition, S8 formed a hydrogen bond with the backbone of αY190. 

### 2.14. SCNTX Bound to α7 nAChR but Not to Muscle-Type nAChR

The experimental data on SCNTX shows that it binds to (α1)_2_β1εδ muscle-type nAChR but not to α4β2 nAChR with no information on α7 nAChR binding. To test whether SCNTX can bind to α7 nAChR, we docked SCNTX to both α7 and muscle-type nAChR structures. SCNTX R31 extended into the aromatic cage of the α7 nAChR and formed interactions with Y188, Y195, and W149. A guanidinium hydrogen formed a hydrogen bond with the H30 of the peptide, which interacted with (−)W55. D29 also formed a hydrogen bond with the neighboring H30. Other important interactions were a hydrogen bond between T33 and E189, and a salt bridge between R28 and (−)E162. The score versus RMSD plots showed funneling behavior, although they were more scattered compared to that of long-chain 3FTX. SCNTX did not bind under the C-loop and only formed weak interactions with αY188 at the αγ and αδ interfaces with no funneling behavior observed at the score versus RMSD plots (Figure 8). 

### 2.15. Oh9-1 Binding to nAChR Was Tested with Two Different Configurations

Oh9-1 belongs to ω-bungarotoxin family and interacts with the nAChR through its aromatic residues rather than charged residues in comparison to α-neurotoxins that form the bulk of the peptides studied in this work [42]. Oh9-1 was previously shown to bind muscle-type nAChR but not α7 nAChR. Because of its structural differences, the way it binds the nAChR is likely different in comparison to α-neurotoxins. In the α7 nAChR and *Torpedo* nAChR αδ interface docking calculations, all Oh9-1 poses were on the outer side of the C-loop with no interactions with the aromatic cage residues. The score versus RMSD plots lacked funneling behavior, further in support of lack of Oh9-1 binding at these two interfaces. 

Oh9-1 formed interactions with the outer face of the C-loop and the F-loop at the αγ and αδ interfaces. At the αδ interface, N30 formed a hydrogen bond with W187, and P32 and F28 formed hydrophobic interactions with Y189 and T191 respectively. At the αγ interface, hydrogen bonds were formed between N30 and γE176, H7 and αY189, and H31 and the backbone of αC192. P32 and αP194 also formed hydrophobic interactions. However, these models lacked interactions between the peptide residues 23–27 and the protein, which were shown to be important in mutagenesis studies [42].

### 2.16. Rotation of the Oh9-1 Starting Poses Affects the Results of the Docking Calculations

**Figure 11:**
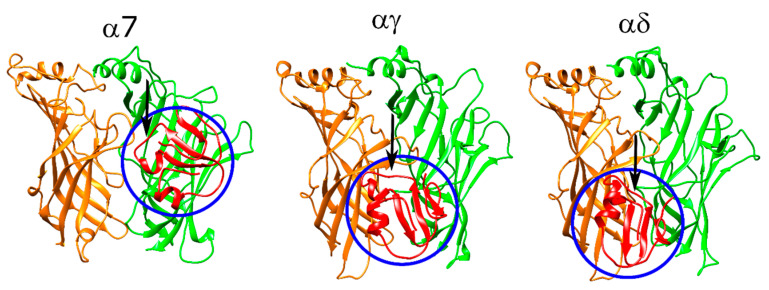
Structures of the lowest-scoring “flipped” Oh9-1-nAChR complexes at the α7 (**left**), αγ (**middle**), and αδ interfaces (**right**). The orange color stands for the positive subunit, the green color stands for the negative (complementary) subunit, and the red color stands for the peptide. The peptide is shown with blue circles and the critical region for Oh9-1 binding to nAChR is shown with an arrow.

Next, the Oh9-1 molecule was “flipped” such that the residues found to be important in experimental studies face the C-loop and the F-loop of the protein. The score versus RMSD plots resulting from these calculations were scattered for the α7 runs but showed funneling behavior for the αγ and αδ runs (Figure S6). The α7 poses had the residues 23–27 mostly sitting on the (−) face of the receptor (Figure 11, left panel). This arrangement placed the F27, F28, and H31 inside the aromatic cage interacting with Y188, Y195, (−)W55, and (−)L109. T25 formed a hydrogen bond with (−)H115, and K46 formed a hydrogen bond with S113. 

For the αγ and αδ interfaces, the peptide interacted with the outer face of the C-loop through hydrophobic interactions. At the αγ interface, F27 and F28 formed hydrophobic interactions with αW187, αP197, αY189, and αV188 (Figure 11, middle panel). T25 interacted with the backbone of γP181, and K46 formed a salt bridge with γE186. At the αδ interface, F28 formed π–π stacking interactions with αW187, and F27 formed such interactions with αY189. K46 interacted with δE164 (Figure 11, right panel). The remaining residues that affected peptide activity did not interact with the receptor but rather interacted with other residues within the peptide, which may be responsible for its structural stability.

### 2.17. Pr-SNTX May Bind to α7 nAChR with Weak Affinity

**Figure 12:**
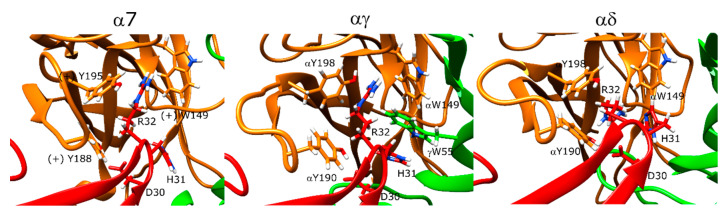
Structures of the lowest-scoring Pr-SNTX-nAChR complexes at the α7 (**left**), αγ (**middle**), and αδ interfaces (**right**). The orange color stands for the positive subunit, the green color stands for the negative (complementary) subunit, and the red color stands for the peptide.

Pr-SNTX inhibited binding to muscle-type nAChR at nanomolar concentrations but showed no inhibitory activity in experiments with α7 nAChR up to a concentration of 10 μM [41]. However, Pr-SNTX was identified as a weak binder to α7 nAChR in our calculations with a stable binding pose that resembled the binding pose of erabutoxin-a (Figure 12, left panel). The R32 of the peptide electrostatically interacted with the aromatic cage residues W149, Y188, and Y195, and the D30 of the peptide formed a hydrogen bond with the -OH group of Y188. In addition to these interactions, N7 formed a hydrogen bond with the backbone of F187, and R35 side chain formed an interaction with Y188 at the outer face of the C-loop. 

Pr-SNTX poses showed funneling behavior at both αγ and αδ interfaces, but the trend was more visible in the former plot. At the αγ interface, R32 extended into the aromatic cage and formed interactions with αY190, αW149, αY198, and γW55. H31 also interacted with γW55. A salt bridge was formed between K46 and γE176 (Figure 12, middle panel). The αδ interface interactions were similar except the R32 extended below the C-loop rather than running parallel to the C-loop, and an additional interaction was observed with δL121 (Figure 12, right panel). 

## 3. Discussion

### 3.1. Rosetta Protein–Protein Docking Can Accurately Model Peptide–Protein Interactions 

Our re-docking and cross-docking calculations showed that Rosetta protein–protein docking can reproduce the binding orientations from crystal structures with RMSD values below 2 Å for the former and lower than 3 Å for the latter case. The following screening calculations with 15 short and long-chain 3FTX accurately predicted the binding of four long-chain 3FTXs to AChBP, whereas poses outside the C-loop or weak interactions with the orthosteric site were observed for the non-binder short-chain 3FTX. 

10 of the 11 non-binder peptides screened for AChBP binding showed no stable poses in contact with the orthosteric site aromatic residues as predicted by the experiments. The only exception was **T16** that formed weak interactions with Y188. Although this peptide was identified as a non-binder in the fishing experiments, a homolog of this peptide with 77% homology (NmmI from *Naja mossambica mossambica*) is known to bind α7 nAChR and muscle-type nAChR [24,48]. Therefore, the predicted **T16** binding in the docking calculations may be a reflection of binding to AChBP, which was not strong enough to be observed under experimental conditions.

### 3.2. Long-Chain 3FTX Have Docking Poses Consistent with Experimental Data 

α-bungarotoxin formed several interactions with the tyrosine resides of the orthosteric site in the muscle-type nAChR. R36 faced upwards where the carbon groups of the residue formed hydrophobic interactions with αY190, and the nitrogen groups formed a hydrogen bond with αY198. Mutation of both these residues resulted in a decrease in α-bungarotoxin binding to muscle-type nAChR in experimental studies [44,45]. F32 formed hydrophobic interactions with γL118 and γW54, known to be important for α-bungarotoxin binding [46]. There was an ~3 Å difference between the coordinates of the α-bungarotoxin molecule in the experimental structure versus the lowest-scoring pose from the docking calculations, but the interaction network was similar between the two structures. 

Experimental studies have identified some of the important residues in α7–α-cobratoxin mutations as Y188F/T/A/R/K, D164K, and W55F [49]. Our results capitulated the importance of Y188 and W55 in α-cobratoxin binding. Although there were no interactions between D164 and the peptide residues, mutation of the aspartate residue to a lysine could cause clashes with the K35 residue, therefore diminish binding. As for the α-cobratoxin residues, F29, R33, K35, and F65 were shown to be important for α-cobratoxin binding to α7 nAChR residues [47,49], which interacted with several protein residues in our docking calculations. 

Drysdalin formed more interactions with the aromatic cage of the muscle-type nAChR interfaces in comparison to α7 nAChR. Both αγ and αδ showed interactions between R30 and αY190 and αY198, whereas only interactions with Y188 were formed at α7. Binding at the αδ interface involved additional interactions between F29 and δW57 and δL121 similar to α-bungarotoxin and α-cobratoxin. On the other hand, α7 had an additional salt bridge between K36 and E189, which was absent in the muscle-type interface poses since the α1 subunit has a threonine residue at this position. The N-terminal residue 68 was observed to interact with the residues on the outer face of the C-loop in all three docking calculations. 

### 3.3. Erabutoxin-a and Pr-SNTX Stably Bind to α7 and Muscle-Type nAChR 

Erabutoxin-a formed stable interactions with the binding sites of α7 and muscle-type nAChR through D31, F32, and R33, all of which were shown to be important for erabutoxin-a binding to the muscle-type nAChR [50]. Pr-SNTX showed no binding to α7 nAChR at concentrations as high as 10 μM under experimental conditions, and therefore, it was originally considered as a non-binder to this protein [41]. However, our results show a binding pose consistent with erabutoxin-a that binds α7 nAChR at high-micromolar concentrations; therefore, Pr-SNTX may target α7 nAChR at higher concentrations. Pr-SNTX binding poses at both α7 and muscle-type nAChR calculations resembled erabutoxin-a. The muscle-type interface calculations had additional interactions between K46 and γE176 and H31 and δL121, which may explain the better binding of Pr-SNTX to muscle-type receptors in comparison to α7 nAChR. 

### 3.4. Candoxin Binding Was Inconsistent with Previous Experimental and Computational Data

Candoxin binding to α7 nAChR showed no convergence to a single stable binding pose, but the analysis of the lowest-scoring structures showed interactions between candoxin and α7 nAChR consistent with candoxin binding to this receptor. However, the observed interactions were too weak to explain the high affinity of candoxin towards α7 nAChR. A high-affinity and a low-affinity binding site on the muscle-type nAChR was proposed for candoxin previously [9]. Our results suggest that higher affinity binding can be achieved at the αγ interface of the receptor. On the other hand, complete lack of binding at the αδ interface in our docking calculations is not consistent with a low-affinity binding site.

Previous protein docking and molecular dynamics (MD) calculations showed stable binding of candoxin to both α7 and the αγ interface of the muscle-type nAChR with similar poses [25]. R35 interacted with the α7 residues W149, S150, S151, and (−)L109 and E33 formed a hydrogen bond with Y195. These residues are different than the residues identified in this study such that candoxin binding to α7 involved interactions with Y188, and binding to the αγ interface involved interactions with αY190, αY198, and αW149. Moreover, E33 formed a hydrogen bond with Y188 (αY190) in both of our models. 

There are several possibilities for the differences between the previous calculations and our calculations. Firstly, the *Torpedo* nAChR receptor used for docking is an older model (PDB ID: 2BG9 [51]), which is of lower resolution, lacks the F-loop, and suffers from magnification errors [18]. Secondly, the docking protocol used in the previous study utilized a single protein conformation and the HEX software for the docking step, different than our ensemble docking approach with Rosetta. Finally, the docked structures were subjected to further MD simulations, which may have changed the binding configuration of the peptides. 

### 3.5. SCNTX Binding to Muscle-Type nAChR Was Not Predicted by the Docking Protocol

SCNTX was tested with α4β2, but not α7 nAChR, in the experimental studies [40]. However, many 3FTX that bind to α7 nAChR have no effect on α4β2, and therefore, these results cannot be extrapolated for SCNTX binding to α7 nAChR. Our calculations with SCNTX showed that this peptide can bind α7 nAChR and form interactions with the aromatic cage residues. Interestingly, SCNTX did not bind under the C-loop and only formed weak interactions with αY188 at the αγ and αδ interfaces despite its known experimental activity at this receptor. The activity of SCNTX was tested on (α1)_2_β1εδ receptor type; however, we used the αγ subunit instead of the αε subunit in our calculations due to the availability of that experimental structure. The lack of αγ and αδ binding would be understandable if SCNTX binds selectively to the αε interface, but there are no experimental data to validate this idea. Further studies including the αε interface will be necessary to understand the nature of SCNTX–muscle-type nAChR interactions. 

### 3.6. ω-Bungarotoxin Oh9-1 May Bind to nAChR Configurations Different than Observed for Long and Short-Chain 3FTX

In the initial docking calculations, Oh9-1 showed no binding to α7 nAChR as observed in the experiments. For the αδ and αγ interfaces, some residues were found to be in close contact with the outer face of the C-loop. Of the residues identified in our calculations, H7, F28, and H31 were observed to be important for binding, but mutation of N30 had no effect [42]. On the other hand, our calculations could not address the reason behind the importance of the residues 23–27. Further, the score versus RMSD plots were scattered with no significant convergence, suggesting that these poses may not represent a stable low-energy binding configuration. 

To test whether placement of the residues in a different configuration would affect the results, we rotated the Oh9-1 structure by ~90° such that the important residues identified in experimental studies faced the C-loop. This flipped configuration gave different results for α7 and muscle-type nAChR interfaces, although only the latter interfaces showed funneling behavior in the score versus RMSD plots. The new interactions were between the aromatic residues of the peptide and the aromatic residues of the outer face of the C-loop. These results are the first to shed light into how the ω-bungarotoxin Oh9-1 may interact with the nAChR at structural level.

### 3.7. Limitations of the Protocol

Despite the overall success of our protocol, there were some limitations that affected its performance. Our protocol showed good success in predicting the native structures of AChBP-bound peptides and qualitative prediction of binders and non-binders of this protein, but its success in nAChR calculations was apparently more limited compared to the AChBP calculations. Three major factors that likely affected the performance of our calculations are artifacts associated with homology modeling, the innate mobility of the C- and F-loops and issues with modeling interactions such as cation–π and π–π interactions. 

### 3.8. Homology Modeling Yielded Models with Structural Variability

Our protocol relied on homology modeling to obtain the structures of the majority of the peptides screened for AChBP binding and drysdalin, Oh9-1, Pr-SNTX, and SCNTX. Structural differences were observed among the low-scoring homology models of these peptides particularly at the loop regions that interact with the orthosteric site, which may reflect the innate mobility of these peptides. Because it is not possible to further improve the quality of our homology models in the absence of experimental data, finding the most representative structure for the peptides remains a problem.

Homology modeling was also done to obtain the structure of the α7 nAChR from α7-AChBP. While the two structures have 64% homology, conformational flexibility of the C- and F-loops of the α7 nAChR structure may have caused issues at the homology modeling stage. Extended loop configurations can be challenging to model with homology modeling. Different low-scoring structures showed variations at two regions suggesting the presence of alternative configurations. The configurations of the C- and the F-loops may be stabilized upon ligand binding, but there is limited structural insight as to how different peptides affect these configurations. 

### 3.9. Mobility of the C- and F-Loops May Drastically Change nAChR Conformations

Although some structural flexibility is introduced during the relax calculations, our approach primarily assumes that long-chain, short-chain, and non-conventional 3FTX bind to the same conformation of the nAChR. However, this assumption may not be realistic. The C- and the F-loops of the nAChR are known to be very mobile and they respond differently to binding of different ligands. The mobility of the F-loop is also evident in the fact that several AChBP structures and an earlier *Torpedo* nAChR structure have a significant number of missing residues in this region, especially in the absence of a bound ligand. The results of erabutoxin-a and Pr-SNTX calculations are suggestive that this is not an issue with all the short-chain 3FTX analyzed. However, lack of funneling behavior in the candoxin calculations and the lack of binding of SCNTX to muscle-type nAChR may be signs of larger-scale structural changes at the F-loop associated with binding of these peptides. 

### 3.10. Lack of Partial Covalent Interactions in the Rosetta Score Function May Affect Correct Assignment of Side Chain Conformations

The differences between the side chains reported in the crystal structures and the Rosetta-predicted structures can be due to two reasons. The first reason is erroneous prediction of side chain coordinates in the crystal structures due to resolution issues. However, there was no correlation between structural factors and the calculated Fnat values in the re-docking calculations, rendering this possibility less likely. The second reason is the shortcomings in the way Rosetta score function handles non-bonded interactions, which do not fully address interactions such as cation–π and π–π stacking that are important for nAChR binding. Although this shortcoming may not be a major issue while modeling protein–protein interactions in general, the aromatic cage of nAChR are known to interact heavily with positively charged groups of both small molecule ligands and peptide ligands. Further, these aromatic residues can also form interactions with the aromatic groups of the peptides observed for both long- and short-chain 3FTX. Therefore, an improved score function with better representation of non-bonded interactions may improve the performance of our docking protocol.

## 4. Conclusions

Our results show that Rosetta protein–protein docking is a useful tool to model the interactions between 3FTX and nAChR. The protocol was validated in calculations that reproduced the geometries of native neurotoxin-bound AChBP with good success and accurately distinguished binder and non-binder peptides to AChBP. Binding of three long-chain and two short-chain 3FTX was predicted with good accuracy, but the results belonging to three peptides showed deviations from experimental results either at the α7 or muscle-type nAChR docking calculations. Further, our docking results showed no clear patterns explaining the selectivity difference of nAChR ligands that selectively target α7 and muscle-type nAChR based on visual inspection of docking poses or simple metrics such as the docking scores. On the other hand, the deviations from the experimental results may be explained by the parameters used in some experiments including peptide concentration and the muscle-type nAChR receptor subtype that might affect the apparent binding properties of these peptides. Overall, this protocol is suitable for screening 3FTX binding to nAChR and AChBP as a starting point in the analysis of these molecules starting only with sequence information, but future studies are necessary to improve its performance such as inclusion of partial covalent interactions to the score function and identification of more precise metrics to measure peptide binding in predicting selectivity profile of 3FTX at different nAChR subtypes. 

## 5. Materials and Methods

### 5.1. Selection of the Crystal Structures for the Re-Docking and Cross-Docking Calculations

AChBP and α7-AChBP crystal structures with bound neurotoxins were selected for the analyses. The set native structures used for the re-docking calculations consisted of α-bungarotoxin[V31A]-bound α7-AChBP [19] and α-cobratoxin-bound AChBP [22] for the long 3-FTX, and α-conotoxins PnIA, ImI, TxIA (A10L), BuIA, GIC, PeIA, LsIA, LvIA (Table 1). 

### 5.2. Homology Modeling of the Venom Peptides for AChBP Binding Calculations

Structures of the peptides were available only for **T9**, **T10**, and **T16**. For the remaining peptides, structures were obtained through homology modeling calculations with RosettaCM [52]. Templates were selected based on pBLAST results with a known PDB structure that had the highest sequence similarity to the target structure (Table S6), and 3- and 9-mer fragments were generated locally using secondary structure predictions made by the PSIPRED server [53,54]. Disulfide files were used to conserve the disulfide pairs of the peptides based on the disulfide bonding scheme of the template structures. A total of 1000 structures were generated for each peptide, and the lowest-scoring structure was used for the docking calculations. 

### 5.3. Modeling of nAChR

α-bungarotoxin-bound *Torpedo* nAChR cryo-EM structure was used as the docking model for the αγ and αδ interfaces of the muscle-type nAChR (PDB ID: 6UWZ) [18]. 

α-bungarotoxin-bound α7-AChBP (PDB ID: 4HQP) structure [19] was used as the template for the homology model of the α7 ECD, and the mature α7 ECD sequence was used as the query sequence (Uniprot ID: P36544, CHRNA7). 3- and 9-mer fragments were generated locally, and the PSIPRED server was used for the prediction of the secondary structure of the target sequence. A total of 1000 structures were generated in total with RosettaCM with enforced symmetry. The resulting models from these calculations showed dissociation of the peptide and closure of the C-loop, so the relax step of the calculation was constrained to the starting template using a cst_weight of 0.3 to keep the C-loop open in the homology model in a second round of calculations. The constrained models showed no peptide dissociation during the modeling procedure. The lowest-scoring structure was selected as the model for the α7 ECD. 

### 5.4. Relax with the Bound Peptides

The peptide geometries were optimized, and the side chains were repacked through two Rosetta relax calculations [55]. In the first step, the starting model was subjected to a fixed-backbone relax calculation to relieve any significant side chain clashes between the peptides and the proteins. In the second step, FastRelax with backbone movement was done to optimize different peptide–protein interactions, and a total of 200 structures were generated. The lowest-scoring 5 structures were selected as the input for the protein–protein docking calculations.

### 5.5. Protein–Protein Docking Calculations

Rosetta protein–protein docking protocol was used for all the docking calculations. The five lowest-scoring structures from the relax step were used as the starting structures for the docking step. The protein chains were kept fixed, and the peptide chain was allowed to move with a step size of 3 Å translation and 8° rotation. A total of 500 structures were generated for each starting point to a total of 2500 docked structures. The five poses with the lowest interface score (I_sc) were inspected visually and analyzed for the binding scores and per-residue scores. 

### 5.6. Analysis of the Docked Structures

The structure with the lowest total score was used as the native structure for the RMSD calculations of 15 peptides to AChBP and the docking calculations of eight peptides to nAChR. The score versus RMSD plots were generated based on these RMSD values. For the native re-docking calculations, the experimental structures were used as the native structure, and values including Fnat, I_sc, Irms, and total RMSD were calculated. For the cross-docking calculations, the resulting cross-docked structures were aligned with the native structure belonging to the peptide–protein complex, and the RMSD values were calculated with UCSF Chimera [56]. Matplotlib module of python was used to generate all the plots.

## Figures and Tables

**Table 1:** Protein Data Bank (PDB) IDs and the bound ligands of the acetylcholine binding protein (AChBP) and α7-AChBP used for the re-docking and cross-docking calculations. *Ls* stands for Lymnaea stagnalis and *Ac* stands for Aplysia californica.

Ligand	Protein	PDB ID
α-bungarotoxin	α7/*Ls*-AChBP	4HQP [19]
α-cobratoxin	*Ls*-AChBP	1YI5 [22]
α-conotoxin PnIA	*Ac*-AChBP	2BR8 [29]
α-conotoxin ImI	*Ac*-AChBP	2BYP, 2C9T [30,31]
α-conotoxin TxIA (A10L)	*Ac*-AChBP	2UZ6 [32]
α-conotoxin BuIA	*Ac*-AChBP	4EZ1
α-conotoxin GIC	*Ac*-AChBP	5CO5 [33]
α-conotoxin PeIA	*Ac*-AChBP	5JME
α-conotoxin LsIA	*Ls*-AChBP	5T90 [34]
α-conotoxin LvIA	*Ac*-AChBP	5XGL [35]

## Supplementary Materials

The following are available online at https://www.mdpi.com/2072-6651/12/9/598/s1, Figure S1: Comparison of the native geometries from PDB (beige) and the best Rosetta docking result (blue), Figure S2: Plots showing the relation between the Fnat values and the percentage of side-chain outliers (left), R-values (middle), and structure resolution (right), Figure S3: Three AChBP structures that show variations in the C-loop or F-loop positions with all five subunits of the structure aligned. The two loops are marked with black arrows, Figure S4: The score versus RMSD plots of the peptides screened for binding to AChBP, Figure S5: T06, T10, T14, and T16 lowest interface score poses from the docking calculations. The loop II of each peptide is circled in red, Figure S6: The score versus RMSD plots of the “flipped” Oh9-1 docking poses to α7, αδ, and αγ interfaces, Table S1: The RMSD values calculated for the five poses with the lowest interface scores for each peptide—protein complex in the native re-docking benchmark set. All units are in Angstroms (Å), Table S2: The RMSD values calculated for the five poses with the lowest interface scores for each peptide—protein complex in the cross-docking benchmark set. The first PDB ID indicates the file from which the peptide was taken from and the second PDB ID indicates the AChBP file into which the peptide was docked. All units are in Angstroms (Å), Table S3: Names of the 3FTX peptides used for the benchmark calculations with AChBP as reported in Albulescu et al. 2019, Table S4: Sequences of the fifteen 3FTX used for the docking calculations with AChBP, Table S5: Sequences of the eight 3FTX used for the docking calculations with α7 and muscle-type nAChR, Table S6: Peptide names and PDB IDs used as the toxin structure or the template.
