# Supplementary material for: An Investigation of Three-Finger Toxin—nAChR Interactions through Rosetta Protein Docking
Source: Toxins (Basel). 2020 Sep 16;12(9):598. doi: 10.3390/toxins12090598 (PMC7551183; doi:10.3390/toxins12090598)
Supplement: Supplementary file 1 [file toxins-12-00598-s001.pdf]

Supplementary Materials: An Investigation of Three-Finger Toxin—nAChR Interactions Through Rosetta Protein Docking

Alican Gulsevin and Jens Meiler

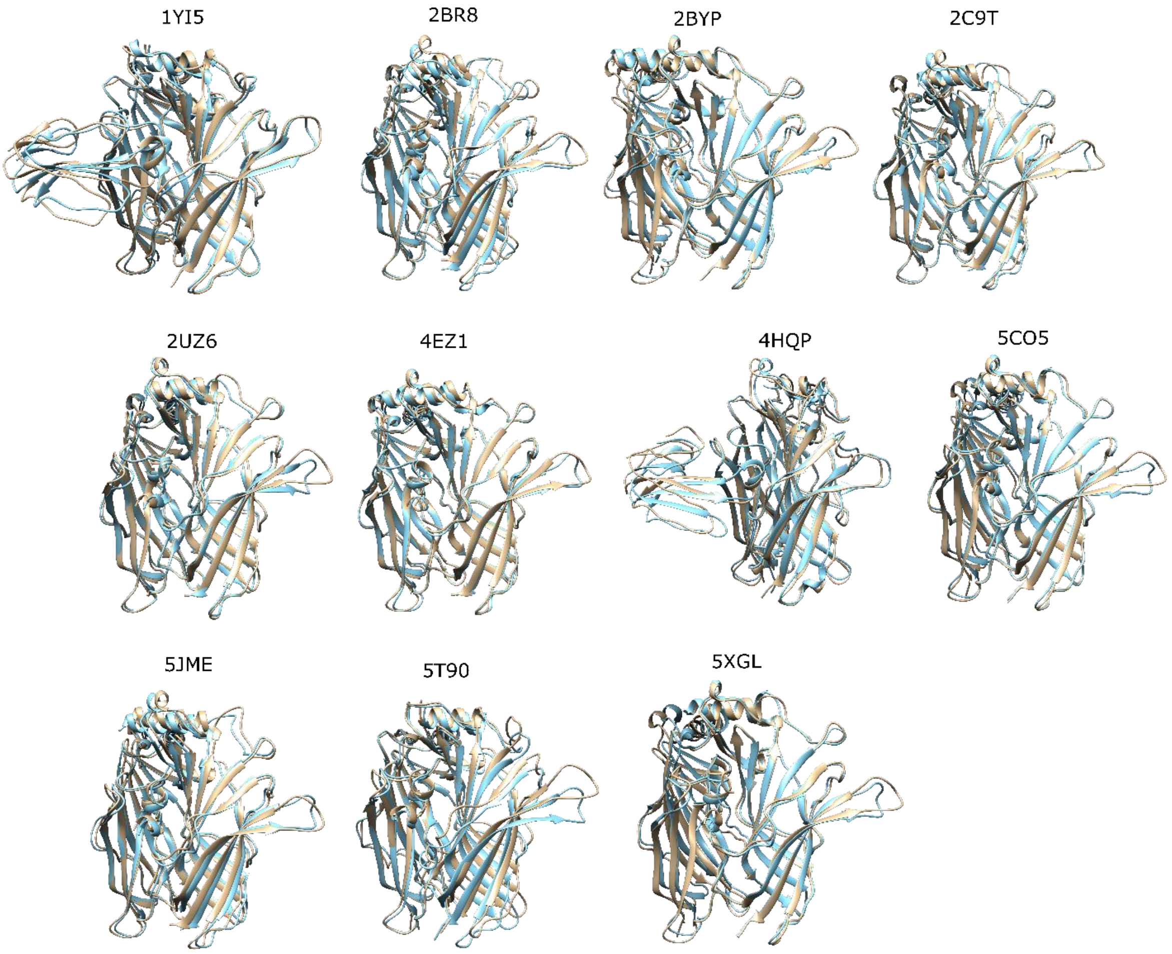

Figure S1. Comparison of the native geometries from PDB (beige) and the best Rosetta docking result (blue).

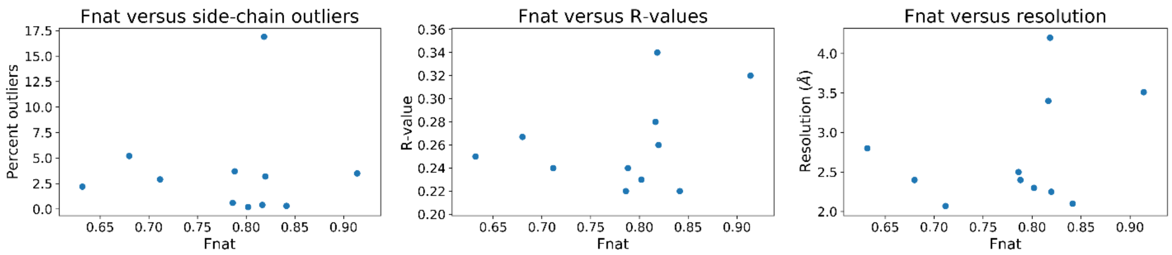

Figure S2: Plots showing the relation between the Fnat values and the percentage of side-chain outliers (left), R-values (middle), and structure resolution (right).

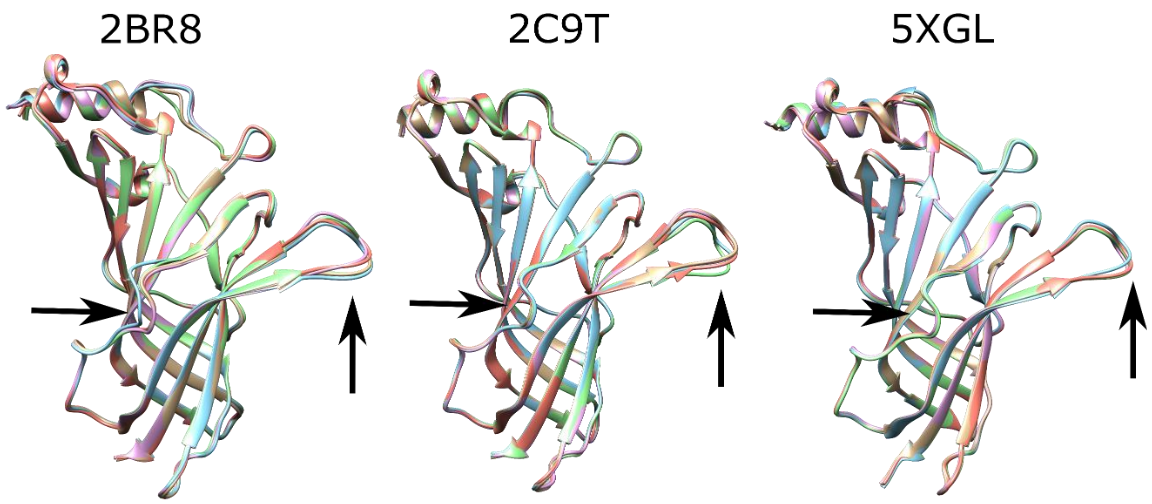

Figure S3. Three AChBP structures that show variations in the C-loop or F-loop positions with all five subunits of the structure aligned. The two loops are marked with black arrows.

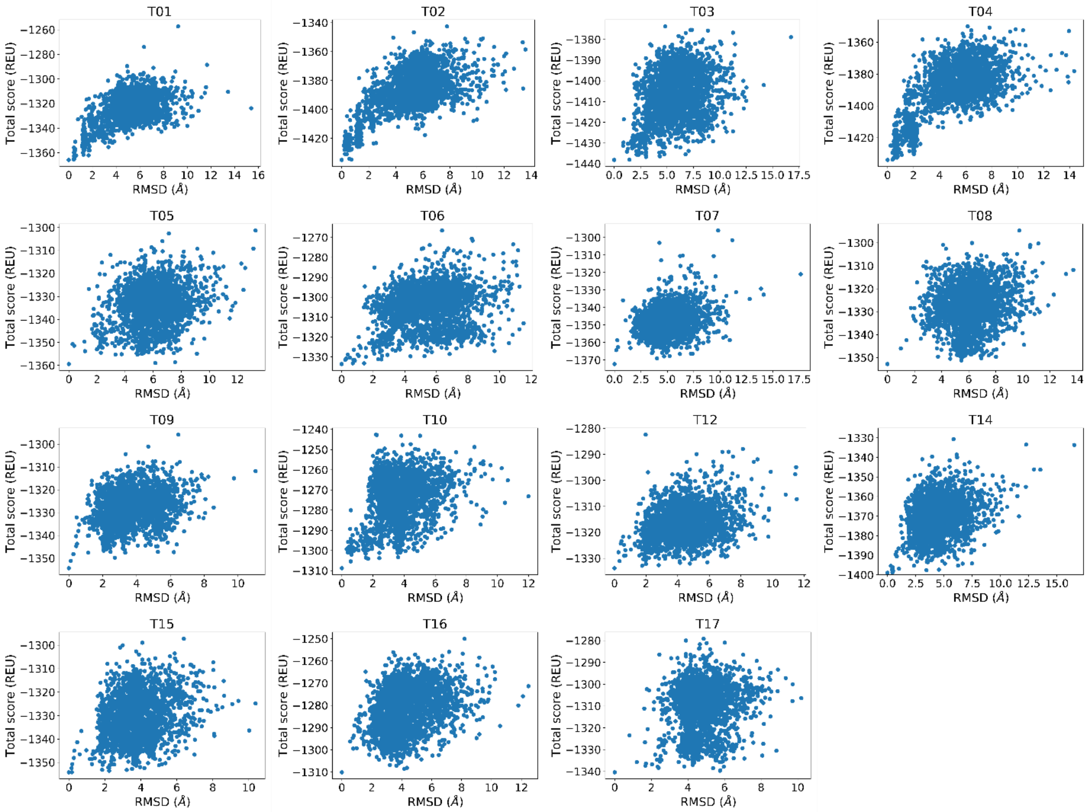

Figure S4: The score versus RMSD plots of the peptides screened for binding to AChBP.

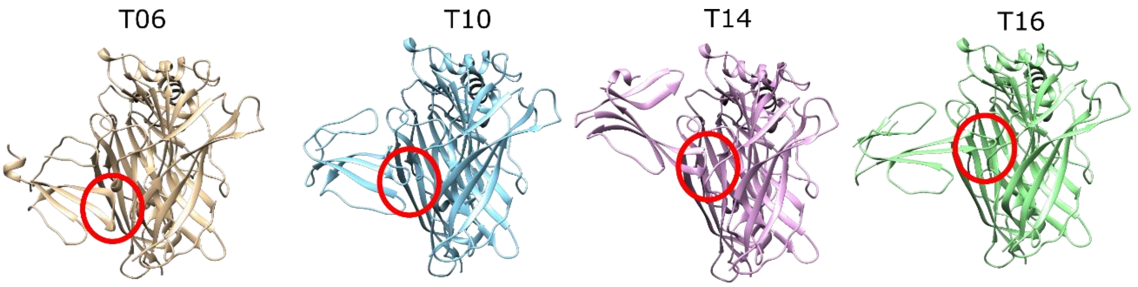

Figure S5: T06, T10, T14, and T16 lowest interface score poses from the docking calculations. The loop II of each peptide is circled in red.

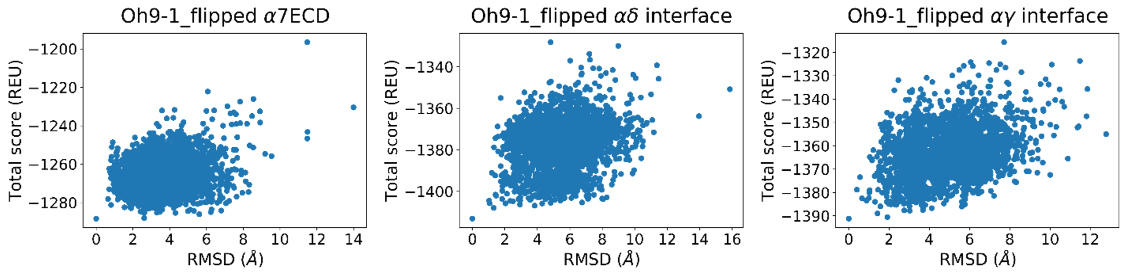

Figure S6. The score versus RMSD plots of the "flipped" Oh9-1 docking poses to α7, αδ, and αγ interfaces.

Table S1: The RMSD values calculated for the five poses with the lowest interface scores for each peptide—protein complex in the native re-docking benchmark set. All units are in Angstroms (Å).

**Pose**	**1YI5**	**2BR8**	**2BYP**	**2C9T**	**2UZ6**	**4EZ1**	**4HQP**	**5CO5**	**5JME**	**5T90**	**5XGL**
1	1.9	1.6	1.8	1.3	1.8	1.4	1.8	1.3	1.5	1.6	1.6
2	1.8	1.5	1.9	1.3	1.8	1.4	1.8	1.3	1.5	1.6	1.7
3	1.9	1.6	1.9	1.3	1.8	1.2	1.6	1.4	1.5	1.6	1.8
4	2.0	1.6	1.9	1.3	1.8	1.4	1.7	1.3	1.5	1.5	1.6
5	1.8	1.5	1.9	1.3	1.8	1.2	1.7	1.3	1.5	1.6	1.6
Average	1.9	1.6	1.9	1.3	1.8	1.3	1.7	1.3	1.5	1.6	1.7

Table S2: The RMSD values calculated for the five poses with the lowest interface scores for each peptide—protein complex in the cross-docking benchmark set. The first PDB ID indicates the file from which the peptide was taken from and the second PDB ID indicates the AChBP file into which the peptide was docked. All units are in Angstroms (Å).

**Pose**	**1YI5_5T90**	**2BR8_2UZ6**	**2BR8_4EZ1**	**2BYP_4EZ1**	**2BYP_5JME**	**2C9T_2UZ6**	**2C9T_5CO5**	**2UZ6_2C9T**	**2UZ6_5XGL**
1	3.1	2.6	1.8	1.7	1.8	1.9	1.3	2.6	1.8
2	3.0	2.7	1.8	1.7	1.8	1.9	1.3	1.8	1.8
3	3.1	2.6	1.8	1.7	1.9	1.9	1.3	1.8	2.2
4	2.3	2.7	1.8	1.7	1.7	1.4	1.2	2.7	1.9
5	2.3	2.6	1.8	1.7	1.7	1.4	1.3	2.0	1.8
Average	2.8	2.6	1.8	1.7	1.8	1.7	1.3	2.2	1.9
**Pose**	**4EZ1_2BR8**	**4EZ1_5JME**	**5CO5_2UZ6**	**5CO5_2BYP**	**5JME_2BR8**	**5JME_5CO5**	**5T90_1YI5**	**5XGL_2BYP**	**5XGL_5JME**
1	2.1	1.5	1.6	1.7	2.2	2.3	2.2	2.2	1.8
2	2.1	1.5	1.7	1.6	2.2	2.2	2.1	2.0	1.8
3	2.1	1.5	1.6	1.6	2.2	2.3	2.2	2.2	1.8
4	2.1	1.5	1.7	1.5	2.2	1.9	2.1	2.1	1.6
5	2.2	1.5	1.7	1.6	2.1	2.0	2.2	2.0	1.8
Average	2.1	1.5	1.7	1.6	2.2	2.1	2.2	2.1	1.8

Table S3: Names of the 3FTX peptides used for the benchmark calculations with AChBP as reported in Albulescu et al. 2019.

**Peptide code**	**Peptide name**
T01	Naja_haje_T4059_T1661_T3904_T2301_3FTX
T02	Naja_haje_T3908_T1873_3FTX
T03	Naja_kaouthia_T1411_3FTX
T04	Naja_naja_T0509_3FTX
T05	Naja_haje_T1255_3FTX
T06	Naja_haje_T1960_T1639_T4413_3FTX

T07	Naja_haje_T2831_T1704_T3906_T3905_T0971_3FTX
T08	Naja_kaouthia_T2210_3FTX
T09	Naja_kaouthia_T5021_T3438_3FTX
T10	Naja_kaouthia_T5505_T3463_3FTX
T11	Naja_kaouthia_T5501_3FTX
T12	Naja_kaouthia_T2473_3FTX
T13	Naja_naja_T1211_T1304_T0704_3FTX
T14	Naja_naja_T1289_3FTX
T15	Naja_naja_T2400_T1263_T0382_T2324_3FTX
T16	Naja_naja_T2381_T2098_T2382_T2412_T2627_T2517_3FTX
T17	Naja_naja_T2420_T2418_T2687_T1672_3FTX

Table S4: Sequences of the fifteen 3FTX used for the docking calculations with AChBP.

T01
MKTLLLTLVLVTILCLDSGYTIRCFITPDVTSQACPDGQNICYTKTWCDNFCGMRGKRVDLGCAATCPTVKPGVDIKCCSTDNCN
T02
MKTLLLTLVLVTILCLDSGYTIRCFITPDVTSQACPAGHVCYTKMWCDNFCGMRGKRVDLGCAATCPTVKPGVDIKCCSTDNCNPFPTRKRS
T03
MKTLLLTLVVVTIVCLDLGYTIRCFITPDITSKDCPNGHVCYTKTWCDAFCSIRGKRVDLGCAATCPTVKTGVDIQCCSTDNCNPFPTRKRP
T04
MKTLLLTLVLVTIVCLDLGYTIRCFITPDITSKDCPNGHVCYTKTWCDGFCRIRGERVDLGCAATCPTVKTGVDIQCCSTDNCNPFPTRKRP
T05
RLCLSDYSIFSETIEICPDGHNFCFKKFPKGITRLPWVIRGCAATCPKAEAQVIVECCTTDKCNR
T06
CLICPEKYCNKVHTCRNGENQCFKRFDQRKLLGKQYRRGCAATCPEAKPREIVECCTTDKCNR
T07
MICHNQQSSQPPTIKTCPGETNCYKKQWRDHRGTIIERGCGCPSVKKGVGIYCCKTDKCNR
T08
RLCLSDYSIFSETIEICPDGHNFCFKKFPKGITRLPWVIRGCAATCPKAEARVYVDCCARDKCNR
T09
LKCNKLVPLFYKTCPAGKNLCYKMFMVATPKVPVKRGCIDVCPKSSLLVKYVCCNTDRCN
T10
LKCNKLIPIASKTCPAGKNLCYKMFMMSDLTIPVKRGCIDVCPKNSLLVKYVCCNTDRCN
T12
LTCLNCPEMFCGKFQICRNGEKICFKKLHQRRPLSWRFIRGCADTCPVGKPYEMIECCSTDKCNR
T14
LKCVKEKSIFGVTTEDCPDGQNLCFKRWHMIVPGRYKKTRGCAATCPIAENRDVIECCSTDKCND
T15
LKCNKLVPLFYKTCPAGKNLCYKMYMVATPKVPVKRGCIDVCPKSSLLVKYVCCNTDRCN
T16
LECHNQQSSQPPTTKTCSGETNCYKKWWSDHRGTIIERGCGCPKVKPGVNLNCCRTDRCNN
T17
LQCNKLVPIASKTCPPGKNLCYKMFMVSDLTIPVKRGCIDVCPKNSLLVKYVCCNTDRCN

Table S5: Sequences of the eight 3FTX used for the docking calculations with $\alpha 7$ and muscle-type nAChR.

α-bungarotoxin
IVCHTTATSPISAVTCPPGENLCYRKMWCDAFCSSRGKVVELGCAATCPSKKPYEEVTCCSTDKCNPHPKQRP
candoxin
MKCKICNFDTCRAGELKVCASGEKYCFKESWREARGTRIERGCAATCPKGSVYGLYVLCCTTDDCN

cobratoxin
IRCFITPDITSKDCPNGHVCYTKTWCDAFCSIRGKRVDLGCAATCPTVKTGVDIQCCSTDNCNPFPTR
drysdalin
RKCYKTHPYKSEPCASGENLCYTKTWCDFRCSQLGKAVELGCAATCPTTKPYEEVTCCSTDDCNRFPNW ERPRPRPRGLLSSIMDHP
erabutoxin
RICFNHQSSQPQTTKTCSPGESSCYNKQWSDFRGTIIERGCGCPTVKPGIKLSCCESEVCNN
Oh9-1
LICHRVHGLLQTCEPDQKFCFRKTTMFFPNHPVLLMGCTSSCPTEKYSVCCSTDKCNK
Pr-SNTX
MICCNQQSSQPKTTTCEGGESSCYKKTWSDHRGSRTERGCGCPHVKPGIKLTCCKTDECNN
SCNTX
MICYNQQSSQPPTTKTCSETSCYKKTWRDHRGTIIERGCGCPKVKPGIKLHCCRTDKCNN

Table S6: Peptide names and PDB IDs used as the toxin structure or the template.

Peptide	PDB ID
T01	1YI5 [1]
T02	1YI5 [1]
T03	1YI5 [1]
T04	1YI5 [1]
T05	4ZQY [2]
T06	2JQP
T07	1IQ9 [3]
T08	4ZQY [2]
T09	1H0J [4]
T10	2CDX [5]
T12	2MJ0 [6]
T14	5MG9 [7]
T15	1H0J [4]
T16	1NOR [8]
T17	2CDX [5]

References

1. Bourne, Y.; Talley, T.T.; Hansen, S.B.; Taylor, P.; Marchot, P. Crystal structure of a Cbtx–AChBP complex reveals essential interactions between snake α-neurotoxins and nicotinic receptors. *EMBO J.* **2005**, *24*, 1512–1522.
2. Barnwal, B.; Jobichen, C.; Girish, V.M.; Foo, C.S.; Sivaraman, J.; Kini, R.M. Ringhalexin from *Hemachatus haemachatus*: A novel inhibitor of extrinsic tenase complex. *Sci. Rep.* **2016**, *6*, 25935.
3. Gilquin, B.; Bourgoin, M.; Ménez, R.; Le Du, M.-H.; Servent, D.; Zinn-Justin, S.; Ménez, A. Motions and structural variability within toxins: Implication for their use as scaffolds for protein engineering. *Protein Sci.* **2003**, *12*, 266–277.
4. Forouhar, F.; Huang, W.N.; Liu, J.H.; Chien, K.Y.; Wu, W.G.; Hsiao, C.D. Structural basis of membrane-induced cardiotoxin A3 oligomerization. *J. Biol. Chem.* **2003**, *278*, 21980–21988.
5. Jahnke, W.; Mierke, D.F.; Béress, L.; Kessler, H. Structure of Cobra Cardiotoxin CTXI as Derived from Nuclear Magnetic Resonance Spectroscopy and Distance Geometry Calculations. *J. Mol. Biol.* **1994**, *240*, 445–458.
6. Lyukmanova, E.N.; Shenkarev, Z.O.; Shulepko, M.A.; Paramonov, A.S.; Chugunov, A.O.; Janickova, H.; Dolejsi, E.; Dolezal, V.; Utkin, Y.N.; Tsetlin, V.I.; et al. Structural insight into specificity of interactions between nonconventional three-finger weak toxin from Naja kaouthia (WTX) and muscarinic acetylcholine receptors. *J. Biol. Chem.* **2015**, *290*, 23616–23630.
7. Blanchet, G.; Alili, D.; Protte, A.; Upert, G.; Gilles, N.; Tepshi, L.; Stura, E.A.; Mourier, G.; Servent, D. Ancestral protein resurrection and engineering opportunities of the mamba aminergic toxins. *Sci. Rep.* **2017**, *7*, 2701.

8. Golovanov, A.P.; Lomize, A.L.; Arseniev, A.S.; Utkin, Y.N.; Tsetlin, V.I. Two-dimensional ^{1}H-NMR study of the spatial structure of neurotoxin II from *Naja naja oxiana*. *Eur. J. Biochem.* **1993**, *213*, 1213–1223.
